# The liver’s dilemma: sensing real danger in a sea of PAMPs: the (arterial) sinusoidal segment theory

**DOI:** 10.3389/fimmu.2024.1503063

**Published:** 2025-01-27

**Authors:** Andrea Henriques-Pons, Natália Vacani-Martins, Carina de Lima Pereira dos Santos, Marcelo Meuser-Batista

**Affiliations:** ^1^ Laboratorio de Inovações em Terapias, Ensino e Bioprodutos, Instituto Oswaldo Cruz, Fundação Oswaldo Cruz, Rio de Janeiro, Brazil; ^2^ Laboratório de Educação Profissional em Técnicas Laboratoriais em Saúde, Escola Politecnica de Saúde Joaquim Venâncio, Fundação Oswaldo Cruz, Rio de Janeiro, Brazil

**Keywords:** liver immunity, hepatic immunological tolerance, danger recognition, arterial sinusoidal theory, PAMP recognition, inflammation

## Abstract

The liver is susceptible to viruses and bacterial infections, tumors, and sterile tissue damage, but immunological danger recognition in the liver is highly unconventional. When analyzing innate and adaptive immunity in the organ, the valid concepts that guide danger recognition and immune response in the periphery should be put aside. In the liver, the vascular anatomy is a game changer, as about 80% of the blood that percolates the organ arrives from the hepatic portal vein, draining blood rich in molecules from the intestinal flora. This 24/7 exposure to high amounts of pathogen-associated molecular pattern (PAMPs) molecules results in hepatic immunological tolerance. In the liver, dendritic, Kupffer (KC), liver sinusoidal endothelial cells (LSECs), and even hepatocytes express PD-L1, a T lymphocyte downregulatory molecule. Most cells express Fas-L, IL-10, TGF-β, low levels of co-stimulatory molecules, lack of or have low levels of MHC-I and/or MHC-II expression. Moreover, other negative regulators such as CTLA-4, IDO-1, and prostaglandin E2 (PGE2) are regularly expressed. Then, how can real danger be discerned and recognized in this sea of PAMPs? This is an open question. Here, we hypothesize that conventional immunological danger recognition can occur in the liver but in specific and minor arterial sinusoidal segments,. Then, in the portal triad, where the hepatic artery ramificates into the stroma and carries arterial blood with no gut-derived PAMPs, there is no evolutive or environmental pressure for immunosuppressive pathways, and conventional immunological danger recognition could occur. Therefore, in arterial sinusoidal segments with no sea of PAMPs, the liver could recognize real danger and support innate and adaptive immunity.

## 1 Introduction

### 1.1 How deep is this sea of PAMPs?

**Figure 1:**
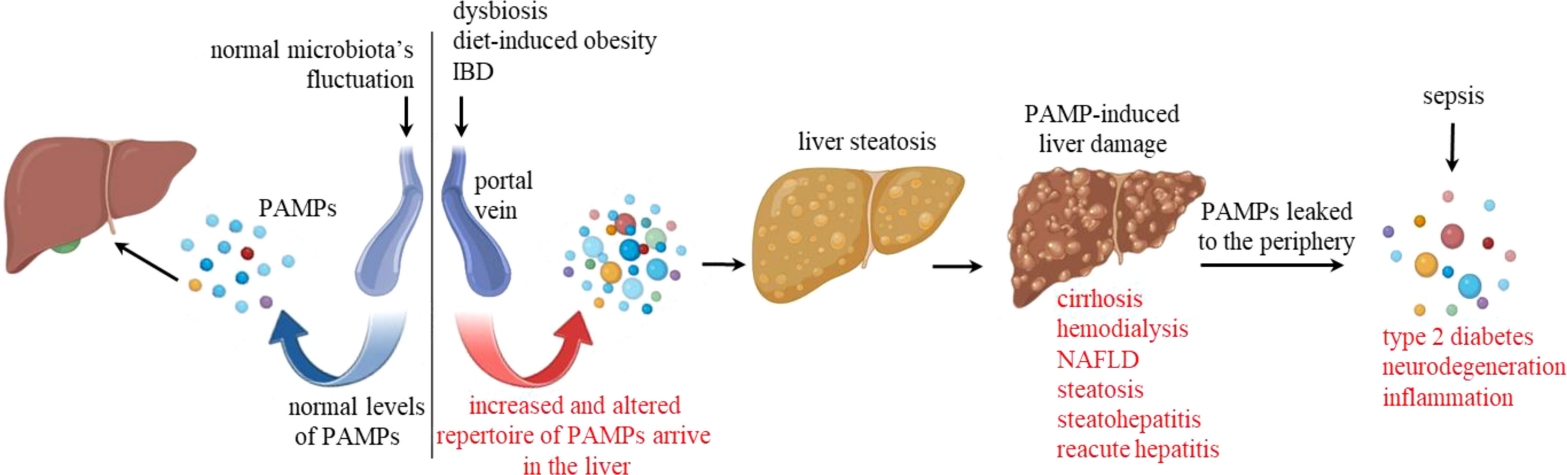
Pathological dysregulation of PAMPs. The left-hand side of the diagram shows normal levels of regular PAMPs, including LPS, entering the hepatic stroma through the portal vein. The right-hand side illustrates upstream pathological conditions leading to increased levels and altered PAMP repertoire in the hepatic portal vein. These conditions include dysbiosis, diet-induced obesity, and inflammatory bowel diseases (IBD). These PAMPs then reach the hepatic stroma and may induce liver steatosis and damage, leading to cirrhosis, hemodynamic conditions that require hemodialysis, nonalcoholic fatty liver disease (NAFLD), steatohepatitis, and reacute hepatitis. When the filtering capacity of the liver is compromised, or in the case of sepsis, higher levels of PAMPs are found in the periphery, leading to inflammatory conditions such as diabetes and neurodegeneration.

The liver blood supply is key to understanding hepatic immunity. It is the only organ with the anatomical peculiarity of having two afferent blood supplies. About 80% of the blood entering the organ is venous blood, brought by the hepatic portal vein. It drains blood from the spleen, stomach, small and large intestines, gallbladder, and pancreas. It is enriched in microbiota’s pathogen-associated molecular pattern (PAMPs) molecules and has low oxygen. The hepatic artery accounts for the remaining 20%, leading well-oxygenated blood to the liver. Usually, endotoxin (LPS) is measured as a marker of intestinal PAMPs in the hepatic portal blood, but the variety of PAMPs is massively higher. The human gastrointestinal tract is inhabited by a complex microbiota community, harboring over 100 trillion microorganisms (1). The microbiota consists of bacteria, protozoa, viruses, archaea, and fungi. Regarding bacteria, the gut microbiota is populated mainly by anaerobic components belonging to Bacteroidetes and Firmicutes phyla. Several environmental and lifestyle factors influence the delicate microbiota’s equilibrium, and the intestinal permeability to those fluctuating levels and repertoire of PAMPs is also variable under no pathological conditions. Therefore, the hepatic stroma must sustain its immunological tolerance to the flora’s components even when exposed to higher amounts of LPS and many other regular and novel PAMPs. On the other hand, conditions leading to a pathological increase of endotoxin levels in the hepatic portal vein usually lead to liver inflammation and damage. These include sepsis (2), inflammatory bowel diseases (IBD) (3), dysbiosis (4), a condition caused by an imbalance in gut microbiota, and diet-induced obesity (1) (**Figure 1**).

However, it is currently unknown, and not discussed here, how the liver could set this differential venous blood “threshold” for intestinal PAMPs as high, although tolerable, or pathologically high and inflammatory when they flow into the organ. On the other hand, the arterial sinusoidal segment hypothesis proposed here aims to contribute to the discussion about how non-intestinal molecular danger can be recognized in the liver. These include protozoans, viruses, infections of non-intestinal bacteria, and tumors. In these cases, danger recognition would be restricted to arterial segments of the sinusoids.

Under normal conditions, the liver maintains its normal physiological functions while filtering a massive and fluctuating amount of PAMPs in venous blood. This interface is essential as peripheral secondary lymphoid tissues would recognize just a fraction of these microbiota bioproducts as proinflammatory immunological danger. Regarding LPS, it is present in the plasma of all healthy humans at variable levels, ranging from 0.01 to 0.5 EU/ml (up to 50 pg/ml) (5). However, endotoxin levels can increase in the peripheral blood due to hepatic pathological conditions that include cirrhosis (6, 7), hemodialysis (8), nonalcoholic fatty liver disease, steatosis, nonalcoholic steatohepatitis (9), chronic hepatitis with acute exacerbation (10) (**Figure 1**). The addition of 10 pg of endotoxin/ml to human blood of healthy individuals is sufficient to activate endothelial cells and monocytes (11), while intravenous injection of 1 ng LPS/kg caused increased blood cytokines (TNF-α, IL-6, IL-8, IL-10), sickness sensation (fatigue, headache, muscle pain, shivering), and reduced motivation within 1 to 3 hours (12). Higher levels of blood LPS in the periphery are therefore associated with increased inflammation, which contribute to atherogenesis (13, 14); increased adiposity and insulin resistance, both of which are precursors to type 2 diabetes (15); neurodegeneration (16) (**Figure 1**); and others. In conclusion, the liver stroma can deal with large amounts of venous LPS and other PAMPs up to a certain point, while the periphery cannot. This hepatic paradigm takes the ‘ ‘liver’s immunological importance to another level.

Under steady-state conditions, LPS and all the other regular and eventual PAMPs enter the liver through the hepatic portal vein and percolate the hepatic lobules through the sinusoids. Multiple anatomical and cellular characteristics in the hepatic stroma potentialize PAMPs clearance, including low blood pressure in the sinusoids and the fenestrae. The resistance to blood flow is extremely low through the liver, with pressure gradients between the portal venous inflow and hepatic venous outflow in the range of 5 mmHg or even less (17). The pressure gradients across all other organs are in the range of 115 mmHg (18). Moreover, the fenestrae allow PAMPs passive diffusion through the sinusoids, favoring their recognition by extravascular cells expressing scavenger receptors for internalization. Liver sinusoidal endothelial cells (LSECs) are important cells for LPS clearance (19), along with Kupffer cells (KCs) (20), and hepatocytes (21). Endogenous endotoxemia is thus likely to be due to reduced clearance of endogenous LPS in portal blood, resulting from decreased scavenger receptor-mediated uptake by the liver’s reticuloendothelial system and parenchymal cells (22).

The literature describes that LPS and other portal PAMPs from the flora (therefore, in venous blood) bind to pattern-recognition receptors (PRRs), such as toll-like receptor 4 (TLR4), with no inflammatory outcome (23). This profile illustrates the immunologically tolerant nature of the liver. For hepatic cells exposed to venous blood, immunological tolerance is coherent, because of the selective pressure of multiple intestinal PAMPs. However, the arterial sinusoidal segments in zone 1 of the hepatic lobules are exposed to no significant levels of PAMPs and, therefore, should be expressing no downregulatory receptors and mediators. These arterial segments are then expected to behave as conventional danger recognition units in the liver.

There is a generalist view in the literature assuming that venous, arterial, and mixed blood sinusoidal segments behave similarly and that all are prone to immunological tolerance in the liver (24). At the same time, it is described that, somehow, in the presence of infections or tumors, hepatic cells “…participate in coordinated immune responses, leading to pathogen clearance, leukocyte recruitment, and antigen presentation to lymphocytes.” (25–27). Moreover, that “…excess(ive) danger molecules presented to TLRs on KCs results in the production of proinflammatory cytokines, including TNF-α, interleukins (IL-1β and IL-6), chemokines (IL-8 and CCL2), and ROS” (28). The inflammatory scenario is presented with no clear mention about what switch could be triggering this response in the liver. The stromal compartmentalization of liver blood segments may be central to understanding this open question.

### 1.2 Mechanisms of liver tolerance

Before we discuss alternative possibilities for danger recognition in the liver, we will revisit the main mechanisms and pathways for liver tolerance. We believe this scenario is restricted to venous and venous/arterial mixed sinusoidal segments. Due to the sinusoid fenestrae, the venous PAMPs reach the adjacent extravascular space (Disse space), favoring their contact with multiple liver cell types (29) that express PRRs and scavenger receptors (30). The fenestrae are the structures by which virtually all resident and flowing intravascular cells make membrane contact. Cellular filopodia are projected through the fenestrae in both directions, with hepatocytes making intimate contact with intra-sinusoidal cells and rolling blood cells contacting the cells in the Disse space and hepatocyte chords (31). The low pressure in the sinusoids favors this enormous and interconnected cellular interaction network and contribute to liver tolerance or inflammation.

#### 1.2.1 Liver endothelial sinusoidal cells

LSECs are fenestrated endothelial cells that line the liver sinusoids; they comprise about 20​% of total liver cells (32) and occupy about 3​% of the liver’s overall volume (33). They secrete prostaglandin E2 (PGE2) and IL-10, which are associated with decreased surface expression of MHC class II, CD80, CD86, mannose receptor activity, and antigen uptake (34). Moreover, they mediate a mechanism of TGF-β-dependent conversion of FoxP3^+^ Treg cells in the liver (35) and their molecular repertoire for immune tolerance includes the programmed death ligand 1 (PD-L1). This molecule binds to PD1 expressed on T cells, and this interaction leads to reduced T cell receptor (TCR), ZAP70, CD28, LCK, and PI3K signaling. Moreover, it reduces cell proliferation, inflammatory cytokines secretion, and T cell survival (36). Other molecules, such as LSECtin, which binds to CD44 (37), and Fas-L prevent T cell activation. Moreover, LSECs naturally fail to produce IL-12; therefore, they typically do not mediate conventional CD4 T cell activation but lead to Treg differentiation (38).

#### 1.2.2 Hepatocytes

The liver parenchyma cells are believed not to express MHC-II under steady-state conditions, although hepatocytes can acquire it during inflammation (39). It was demonstrated *in vitro* that isolated hepatocytes interact with hepatic NK cells through the CD94/NKG2A receptor, leading to dendritic cell- and PD1-mediated Treg cell differentiation (40). Moreover, hepatocytes express low levels of MHC-I and co-stimulatory molecules, such as CD28, while expressing PD-L1. This molecular repertoire is associated with antigen-specific CD8^+^ T cell exhaustion. These dysfunctional lymphocytes result from prolonged or excessive (41) and high (42) antigenic stimulation, plus no optimum co-stimulation or cytokine signaling. Therefore, in chronic viral infections, for example, hepatocytes provide the initial conditions for cytotoxic T lymphocyte activation, but in the absence of the required condition for antigen presentation, the CD8^+^ T cells become dysfunctional and die by apoptosis (43).

#### 1.2.3 Dendritic cells

Hepatic dendritic cell (DC) subsets include plasmacytoid DC (pDC) and conventional DC (cDC), which are subdivided into cDC1 and cDC2 (44). Hepatic DCs are distinct from conventional extrahepatic DCs in multiple aspects (45). They generally express low levels or lack MHC-II and co-stimulatory molecules such as B7-1, B7-2, and CD40. Hepatic DC express high levels of PD-L1 and TGF-β, with lower levels of IFN-γ and more IL-10 than IL-12, favoring Th2 responses and Treg cell differentiation (46, 47). Hepatic DCs express indoleamine 2,3‐dioxygenase 1 (IDO1), an enzyme that plays a role in the degradation of L-tryptophan in downstream kynurenines. Although IDO1 also suppresses immunity through a non-enzymatic activity (48), the accumulation of kynurenine with amino acid depletion is a very efficient mechanism for immunosuppression (49).

#### 1.2.4 Kupffer cells

KCs are hepatic macrophages found in the lumen of the sinusoids. They almost entirely descend from the erythro-myeloid progenitors of the yolk-sac wall. Moreover, blood monocyte-derived KCs are estimated to be about 10% of the total liver population of macrophages (50). They express many immune downregulatory molecules (51), including PD-L1, Fas-L, and IDO1. PRR-independent immunosuppressive pathways include gondoic acid, a monounsaturated omega-9 fatty acid found in various plant oils. It inhibits reactive oxygen species (ROS) production by KCs and reduces inflammation by inhibiting the PKCθ/ERK/STAT3 pathway (52). Moreover, bone marrow stromal cells enhance IL-10 production in KCs via PGE2, which inhibits the NOD-, LRR-, and pyrin domain-containing protein 3 (NLRP3) inflammasome and reduces LPS-induced inflammation (53). KCs secrete IL-10, TGF-β, and PGE2 (54), express low levels of MHC-II, B7-1, B7-2, and CD40, and favor the differentiation of Treg cells (51).

However, like other tissue-resident macrophages, they are apparently divided into M1 and sub-populations of M2 in the liver. M1 KCs secrete inflammatory cytokines such as IL-1β, TNF, and IL-6, while M2 KCs promote tissue repair and angiogenesis. It has been demonstrated that M0 KCs polarization includes the TLR4/NF-κB, JAK/STAT, TGF-β/Smads, PPARγ, and Notch pathways, besides miRNAs and others (55). Then, the recurrent open question remains, which is how hepatic M1 KCs could be differentiated in such a millie rich in IL-10, PGE2, TGF-β, and others?

**Figure 2:**
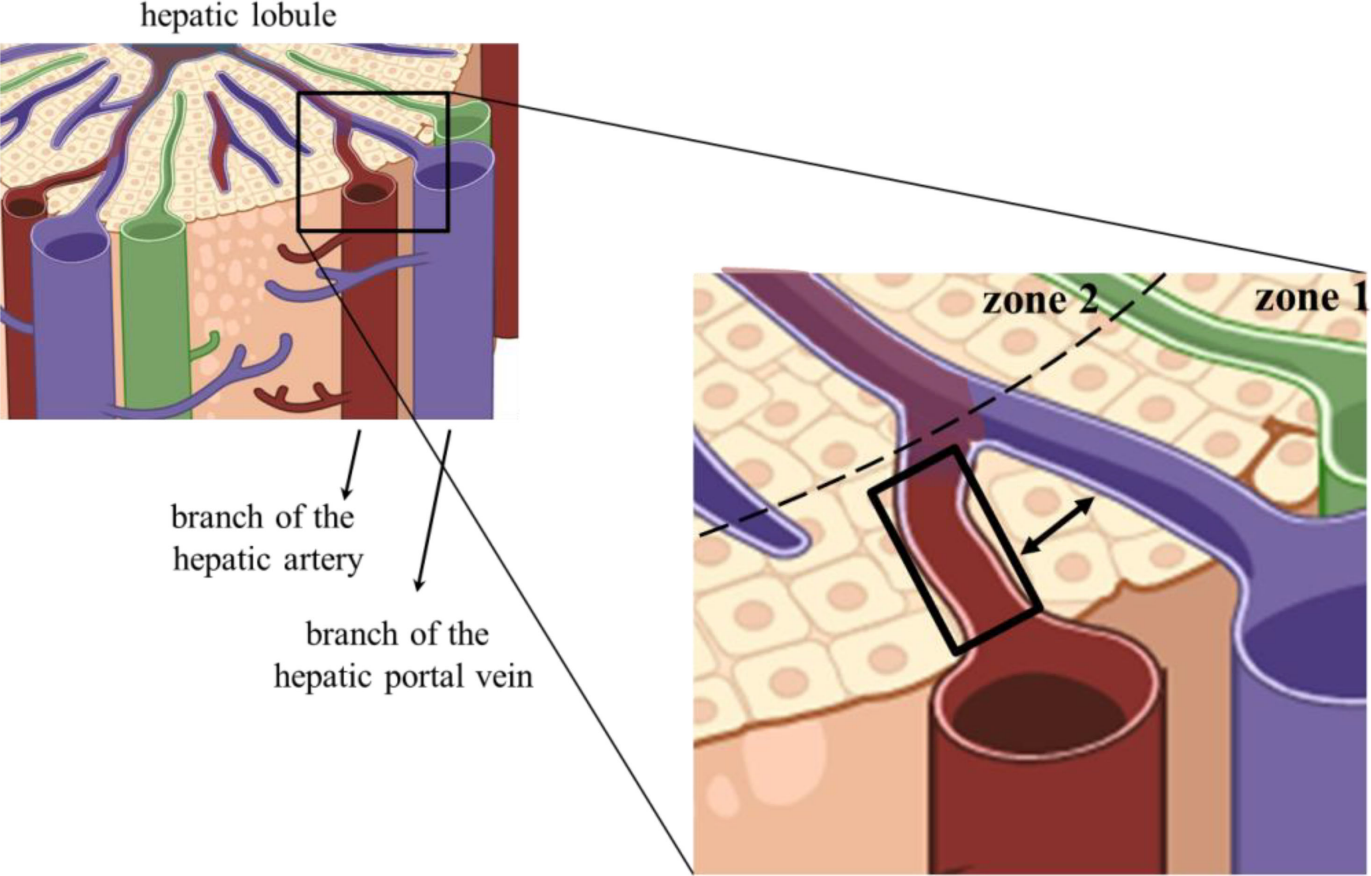
Anatomic location of arterial sinusoidal segments. The left image represents part of a hepatic lobule, and the selected quadrangular area of the portal triad is enlarged on the right-hand side. The black rectangle delimited an arterial sinusoidal segment proposed to harbor hepatic cells bearing no immunosuppressive receptors or cytokines. The double-headed arrow shows that one or two chords of hepatocytes usually separate venous and arterial Disse spaces.

Although not experimentally analyzed yet, M0/M1 or tolerogenic M2 KCs may be anatomically distributed in sinusoidal arterial or venous blood segments, respectively. Similarly, other cell types were described to have functional and phenotypic differences according to their distribution in the liver stroma. It has been described that LSECs display different phenotypes and possibly metabolic functions according to the zonal distribution in the lobule (56). Hepatocytes are also metabolically different along the lobule axe, with sub-populations of hepatocytes having higher activity of gluconeogenesis and fatty acid oxidation in the periportal area, with elevated glycolysis and drug metabolism in the pericentral area (57). This distribution of cellular subpopulations according to the lobule zones is not new, as the term liver “zonation” referring to hepatocyte heterogeneity was coined by Deane in 1944 (58). Here, we propose a more precise cellular anatomic location that does not consider just the lobule zones but also the arterial or venous branches of the sinusoids in those zones. Then, subpopulations of LSECs, hepatocytes, KCs, and other cells would be zonally distributed in arterial or venous blood beds. Tolerogenic KC subpopulations could be enriched in the venous segments in the portal area (zone 1), and mixed venous/arterial blood found in zones 2 and 3. On the other hand, resident M0/M1 KCs and arterial blood monocyte-derived KCs would be enriched in hepatic artery segments in the portal area (zone 1) (**Figure 2**). In these arterial sinusoidal segments, the cells would find an inflammation-permissive environment, with no PAMPs exposure or immunosuppressive pathways (51). However, this possibility remains to be experimentally challenged (59, 60).

### 1.3 Arterial sinusoidal segment theory

We propose that the immunological rules that govern conventional peripheral antigen presentation and immunological danger recognition be applied in the liver’s restricted hepatic segments of arterial blood beds. Therefore, hepatic cellular populations would be empowered in these particular niches to initiate inflammation. Although conciliatory, this proposal has no simple implications. Would antigen-presenting cells (APCs) recognize self-hepatic danger-associated molecular pattern (DAMPs) in these arterial segments? Due to the fenestrae and the low sinusoidal pressure, venous PAMPs could “contaminate” the Disse spaces of arterial segments? How would the molecular immunosuppressive pathways of venous and mixed segments counterbalance the eventual spread of cellular inflammation initiated in the arterial segments?

The major venous blood supply to the liver was evolutionarily necessary to impose a filter barrier before the highly inflammatory PAMPs reached the periphery. Then, the consequent high amounts of PAMPs in the hepatic stroma led the liver to a required tolerant bias. Considering so many soluble and membrane-bound immunosuppressive mediators, it is hard to imagine that they could be anatomically side-by-side with immune danger recognition and cellular inflammation. In the literature, the hepatic tolerance mechanisms are well described and explored. At the same time, once an inflammatory response is established, the inflammatory pathways are also described. There is always this gap as to how inflammation really started. One limitation to understanding this central issue is to consider that all liver cells are exposed to PAMPs, which is not the case. In the arterial sinusoidal segments, with no gut-derived PAMPs, there is no evolutive or immediate selective pressure for expressing immunologically tolerant pathways. This directly implies that LSECs, KCs, hepatic DCs, and stellate cells are not expected to express PD-L1, Fas-L, or all the other downregulatory mediators in the limited arterial sinusoidal segments.

Could it be possible that venous PAMPs traverse the hepatocyte barrier and reach the niche of the arterial segment? This is not expected, although arterial and venous branches are frequently separated by just a layer of hepatocytes (**Figure 2**). Despite the fenestrae and low blood pressure in the sinusoids that facilitate diffusion of blood components to the adjacent Disse spaces, the hepatocytes supposedly prevent two sinusoid segments from sharing their content. Hepatocytes are linearly arranged, forming the hepatocyte chords. They radiate to form a continuous three-dimensional meshwork like spokes on a wheel with one or two layers thick (61) (**Figure 2**). Therefore, considering the 3-D arrangement of the hepatic lobules, one could raise the possibility of venous and arterial blood being mixed due to this very thin layer of hepatocytes as a barrier. However, hepatocytes are believed to be a very efficient barrier. They are highly polarized cells, and the same cells in the chord can be bathed by arterial blood on the apical side and venous blood on the other side. Yet, no blood or stromal soluble components traverse the chords. Inter hepatocyte cell junctions include tight and adherens junctions, desmosomes, and gap junctions (62).

Hepatocytes have a uniquely organized polarity, with a basal membrane facing liver sinusoidal endothelial cells. This polarity is essential for many hepatocytes’ functions and requires coordinated activity of cell adhesion molecules, cell junctions, cytoskeleton, extracellular matrix, and intracellular trafficking machinery. Moreover, establishing and maintaining hepatocyte polarization requires energy, and abnormal functioning may result in severe liver disease (63).

In the arterial segments, we believe APCs can uptake peripheral blood antigens for T cells’ conventional activation. However, considering the hepatic stroma as a whole, and not just the arterial segment niches proposed here, the liver was described as the only non-secondary lymphoid tissue to sustain CD4 and CD8 T cell activation (64, 65). However, this process leads to dysfunctional T cell response, anergy, or Treg differentiation in the organ (66, 67). This dysfunctional activation is valid for most of the hepatic environment but is not aligned with what is expected in arterial PAMP-free sinusoid segments. In these segments, conventional T cell activation would then be possible.

Different from the generalist view of the liver as an immune-tolerant organ, hepatic innate and adaptive immunity would be compatible with the cellular and molecular configuration of the arterial segments. For example, in the case of local infection, antigen presentation by KCs and DCs could be possible by resident arterial segment APCs acting via a conventional immunological synapse. In this case, employing MHC-I and II, co-stimuli, and soluble factors such as IL-1 and IL-12, with no participation of PD-L1, Fas-L, LSECtin, IL-10, TGF-β, or others, that would be restricted to venous segments. Since subpopulations of hepatic DCs and KCs can derive from blood monocytes (68), arterial segments could be further populated by newly differentiated cells, increasing conventional danger recognition and antigen presentation for T-cell priming. Finally, it is known that conventional antigen presentation occurs in secondary lymphoid tissues for liver antigens circulating outside the organ. Then, T cells activated in peripheral secondary lymphoid tissues could also migrate to the non-tolerogenic arterial sinusoidal segments, participating and promoting hepatic immunity.

### 1.4 Migration of activated peripheral T cells to the liver and immune response - not so simple

Experimental evidence supports that peripheral activated/memory T cells accumulated in the liver’s periportal field (69) and promoted a partial shift towards an inflammatory milieu, counterbalancing its natural general tolerance. Although the authors did not evaluate the T cells’ location in sinusoidal portions, the arterial segments are periportal, located in zone 1 (**Figure 2**).

Others have proposed that the induction of T-cell tolerance may depend on whether or not the antigen is first encountered within the liver. In this competition scenario among antigen-presenting sites, an antigen first presented in the liver would lead to tolerance. On the other hand, peripheral activation of T cells would lead to hepatic immune response (70). This peripheral activation and subsequent migration of functional T cells to the liver was also suggested for HCV-specific CD8^+^ T cells (71). Most studies that propose the peripheral activation of T cells for liver immune response use a transfection method aiming to have liver-restricted antigen expression. However, eventual antigen presentation in extrahepatic secondary lymphoid tissues cannot be unequivocally ruled out.

It is apparently obvious that antigens presented in secondary lymphoid tissues lead to T-cell activation and their subsequent migration to primary sites of infection. In this case, to the liver. This is just common ground for immunity in all tissues, but if the site of infection is the liver, things are not so Cartesian. In the liver, already activated T cells can undergo clonal deletion, anergy, deviation, exhaustion, or become dysfunctional (72, 73). Therefore, it is probably not just the case of migrating to the liver. Extrahepatically activated peripheral T cells should then migrate to the right site, or the right sinusoidal segment, in the liver. T cells flowing into the hepatic sinusoids through arterial segments would find a completely different environment than those flowing through venous segments. Either for antigen-primed T cells in secondary lymphoid tissues or naïve T cells for antigen presentation in the arterial segments of the liver.

### 1.5 Activated peripheral T cells contribute to inflammation in the liver

To avoid the previous approaches based on a supposedly restricted antigen expression to the liver, we used a system of adoptively transferred activated T cells. For that, we intraperitoneally injected *Trypanosoma cruzi* extract plus Al(OH) into GFP mice. Then, after boosting, primed CD4^+^ and CD8^+^ splenic T lymphocytes (CD3^+^ CD62L^-^ CD44^high^ CD197^-^) were purified and injected into syngeneic recipient mice that orally received cognate parasite extract. Therefore, the origin of the activated GFP^+^ T cells was undoubtedly peripheral. Another group of mice received just the oral dose of extract. In this group, despite the mucosal exposure to multiple PAMPs, the liver stromal cells responded by reinforcing their tolerogenic pathways. The oral extract increased PD-L1, CTLA-4, IL-10, and TGF-β expression compared to the mice receiving only oral endotoxin-free PBS (74). In a third group, a single intraperitoneal injection of parasite extract plus Al(OH) led to a modest but detectable different scenario in the liver, with increased effector/effector memory intrahepatic T cells (CD3^+^ CD44^high^ CD127^−/+^ CD62L^−^ CD197^−^), PD1 down-regulation, and increased IL-10, TNF, and IL-6. The same pool and amount of antigen extract administered through different routes elicited opposing responses in the liver.

In the first group, when mice received oral extract plus purified GFP^+^ activated T cells, the transferred cells were recovered from the recipients’ perfused liver, confirming that they migrated to the organ. Moreover, these cells induced the accumulation of GFP^-^ CD4^+^ and CD8^+^ T cells in the liver with a phenotype compatible with effector or effector memory T cells. Moreover, there was a reduction of NKT, FoxP3^+^ Treg, and γδ T cells, reduced stromal expression of PD-L1 and CTLA-4. F4/80^+^ KCs increased in the hepatic stroma along with the proinflammatory cytokines TNF, IFN-γ, IL-6, and MCP-1 (74). Our results showed that the transferred peripheral activated T cells migrated to the liver and induced a shift towards a more inflammatory environment in the organ. Since the T-cell transfer impacted different lymphoid and myeloid cell populations, cytokines, and immunoregulatory molecules, those peripheral T cells seemed to have triggered the liver stroma to respond to the danger. Unfortunately, at that time, we did not evaluate if the adoptively transferred T cells in the liver were accumulated in arterial sinusoidal segments, which remains to be studied. The transition of quiescent arterial segments into an immune-activated state for T cell priming would conventionally depend on the presence of processed antigens and danger recognition. Then, intrahepatic immunity would propagate, with activated T cells triggering local inflammation, as observed in the adoptive transfer assays (74).

## 2 Concluding remarks

The arterial sinusoidal segment proposal is compatible with multiple aspects of the hepatic tolerance extensively described in the literature and with the liver’s proven capacity to sustain inflammation and immunity. The arterial segments are limited compared to those enclosing venous and mixed blood, both rich in PAMPs. It is, therefore, compatible with the predominant tolerance bias of the organ. Considering the sinusoidal niches, the liver would be anatomically divided into specific sinusoidal portions dedicated to opposing immunological responses. In arterial segments, hepatic cell subpopulations would express conventional proinflammatory receptors and functional downstream signaling pathways. On the other hand, segments conducting venous/mixed blood would account for most segments of the 3D sinusoidal network in the liver and lead to T cell dysfunction and immune tolerance.

Although it is premature to consider possible clinical applications of the arterial segment theory, we can predict the cellular distribution in anatomic sites for some pathologies initiation. Venous segments would be expected to favor liver cancer and infection, for example, due to T cell silencing and immune downregulation. On the other hand, liver autoimmunity could be more associated with non-venous segments. For intrahepatic T cell priming, rolling T cells must contact APCs in the arterial segments long enough to become fully activated and alter the liver’s immunological equilibrium.

In light of the arterial segment theory, we can speculate about the consequences of liver damage. If it is sufficient to disrupt arterial and non-arterial sinusoidal segments (some examples are shown in **Figure 1**), the stromal and sinusoidal cells in the affected area would be indiscriminately exposed to mixed blood. In this case, arterial segments, their adjacent Disse spaces, and “arterial” hepatocytes would contact intestines-derived PAMPs from the disrupted venous portions. Since arterial segments would recognize immunological danger conventionally, liver inflammation would be expected. On the other hand, depending on the extent of damage, this inflammatory response may not be as exuberant as in other organs. To our knowledge, the proportion of arterial versus venous/mixed segments was not determined; however, the inflammatory arterial segments are just a fraction of the sinusoidal 3D network.

The arterial segment theory offers an underlying explanation for immunological danger recognition in the liver, allowing hepatic cells to initiate inflammation. After all, in the arterial sinusoidal segments, the hepatic cells would be contacting no sea of PAMPs.

## Data Availability

The original contributions presented in the study are included in the article/supplementary material. Further inquiries can be directed to the corresponding author/s.
